# Treatment modalities and drug survival in a systemic sclerosis real-life patient cohort

**DOI:** 10.1186/s13075-020-2140-3

**Published:** 2020-03-23

**Authors:** S. Panopoulos, Κ. Chatzidionysiou, M. G. Tektonidou, V. K. Bournia, A. A. Drosos, Stamatis-Nick C. Liossis, T. Dimitroulas, L. Sakkas, D. Boumpas, P. V. Voulgari, D. Daoussis, K. Thomas, G. Georgiopoulos, G. Vosvotekas, Α. Garyfallos, P. Sidiropoulos, G. Bertsias, D. Vassilopoulos, P. P. Sfikakis

**Affiliations:** 1 Joint Rheumatology Program, 1st Department of Propedeutic Internal Medicine-Rheumatology Unit, National and Kapodistrian University of Athens, School of Medicine, Laikon General Hospital, 17 Agiou Thoma str., 115 27 Athens, Greece; 2 Rheumatology Clinic, Department of Internal Medicine, Medical School, University of Ioannina, Ioannina, Greece; 3 Division of Rheumatology, Department of Internal Medicine, Patras University Hospital, Medical School, University of Patras, Patras, Greece; 4 4th Department of Internal Medicine, Hippokration General Hospital, Medical School, Aristotle University of Thessaloniki, Thessaloniki, Greece; 5 Department of Rheumatology, Faculty of Medicine, School of Health Sciences, University of Thessaly, Larissa, Greece; 6 Joint Rheumatology Program, 4th Department of Internal Medicine, National and Kapodistrian University of Athens, School of Medicine, Attikon University Hospital, Athens, Greece; 7 Joint Rheumatology Program, Clinical Immunology -Rheumatology Unit, 2nd Department of Medicine and Laboratory, National and Kapodistrian University of Athens, School of Medicine, Hippokration General Hospital, Athens, Greece; 8 1st Department of Medicine, Aristotle University of Thessaloniki, School of Medicine, University General Hospital of Thessaloniki AHEPA, Thessaloniki, Greece; 9 Department of Clinical Rheumatology, Clinical Immunology and Allergy, Faculty of Medicine-University of Crete, Heraklion, Greece

**Keywords:** Systemic sclerosis, Treatment patterns, Drug survival, Cohort study

## Abstract

**Background:**

European data indicate that systemic sclerosis (SSc)-related death rates are increasing, thus raising concerns about SSc’s optimal management. Herein, we describe current treatment modalities and drug survival in a real-life SSc cohort.

**Methods:**

Details on immunosuppressive/antiproliferative (methotrexate, mycophenolate, cyclophosphamide, azathioprine, rituximab, tocilizumab) and vasoactive agent [(endothelin receptor antagonists (ERAs), sildenafil, iloprost, and calcium channel blockers (CCB)] administration during the disease course (11.8 ± 8.4 years, mean + SD) of 497 consecutive patients examined between 2016 and 2018 were retrospectively recorded. Drug survival was assessed by Kaplan–Meier analysis.

**Results:**

Methotrexate was the most frequently administered immunosuppressive/antiproliferative agent (53% of patients), followed by cyclophosphamide (26%), mycophenolate (12%), and azathioprine (11%). Regarding vasoactive agents, CCB had been ever administered in 68%, ERAs in 38%, iloprost in 7%, and sildenafil in 7% of patients; 23% of patients with pulmonary fibrosis had never received immunosuppressive/antiproliferative agents, 33% of those with digital ulcers had never received ERAs, iloprost, or sildenafil, whereas 19% of all patients had never received either immunosuppressive/antiproliferative or other than CCB vasoactive agents. Survival rates of methotrexate, cyclophosphamide, and mycophenolate differed significantly, being 84/75%, 59/43%, and 74/63% at 12/24 months, respectively, with inefficacy being the most frequent discontinuation cause. Conversely, CCB, ERAs, and sildenafil had high and comparable retention rates of 97/91%, 88/86%, and 80/80%, respectively.

**Conclusions:**

Existing therapeutic limitations indicate that more evidence-based treatment is warranted for successful management of SSc. Vasculopathy seems to be managed more rigorously, but the low retention rates of immunosuppressive/antiproliferative drugs suggest that effective and targeted disease-modifying agents are warranted.

## Background

Systemic sclerosis (SSc) is a rare systemic autoimmune disorder that pathogenetically encompasses microvascular damage and fibrosis of the skin and visceral organs, associated with immunological aberrations [1] and presents the highest disease-related mortality among the various rheumatic diseases [2, 3]. Despite the emergence of new therapeutic agents for SSc, mainly targeting inflammatory and vascular pathways, survival has not improved significantly during the last decades [4, 5]. Recently, a study from the EUSTAR group reported that despite a decrease in standardized mortality rate in SSc over time, the rate of deaths directly attributed to SSc has increased [6]. Overall, this lack of improved survival raises concerns about the efficacy of currently available treatments and conveys the need for new, more effective agents.

The objectives of the present study are to describe current treatment modalities in a large, multicentre, real-life SSc cohort and to determine the drug survival rate of commonly used immunosuppressive/antiproliferative or vasoactive agents in SSc that reflects their real-world effectiveness and safety.

## Methods

Based on a standardized protocol for data recording procedures and definition of organ involvement, consecutive patients from 8 academic centres across Greece who fulfilled the 1980 American College of Rheumatology classification criteria for SSc [7] and were examined at least once between January 2016 and December 2018, were included in the study. Ethical approval was provided by the Joint Rheumatology Program (Hippokration General Hospital, 64/16-4-2015) as well as by the local institutional boards of participating centres. Informed consent was provided by all patients at baseline. The medical records of all patients were retrospectively analyzed and demographics, SSc clinical manifestations, and any immunosuppressive/antiproliferative or vasoactive treatments administered anytime during the disease course were recorded.

Organ involvement was defined in all participating centres according to the following criteria:

1. Arthritis: presence of synovitis in a joint.
2. Pulmonary fibrosis (PF): usual interstitial pneumonia (UIP) or non-specific interstitial pneumonia (NSIP) pattern on high-resolution chest computed tomography (CT).
3. Pulmonary arterial hypertension (PAH): mean pulmonary artery pressure (PAP) ≥ 25 mmHg on cardiac catheterization or systolic PAP > 50 mmHg in echocardiography.
4. Cardiac rhythm disorders: presence of arrhythmias or conduction defects in ECG confirmed by 24 h Holter monitoring.
5. Renal crisis: Abrupt onset of rapidly progressive oliguric renal failure that cannot be attributed to any other cause with or without concurrent accelerated arterial hypertension (> 150/90 mmHg)
6. Digital ulcers (DUs): denuded area with well-defined borders, involving loss of dermis, epidermis, and subcutaneous tissue located on the volar surface of the fingers.
7. Upper gastrointestinal tract involvement: distal esophageal dysmotility by manometry, dilatation of oesophagus in chest CT, or reported symptoms like heartburn or dysphagia.

Immunosuppressive/antiproliferative agents that were recorded included methotrexate (MTX), mycophenolate mofetil (MMF), cyclophosphamide (CYC), azathioprine (AZA), rituximab (RTX), and tocilizumab (TCZ), while recorded vasoactive therapies comprised endothelin receptor antagonists (ERAs) bosentan, abrisentan, and macitentan, the 5-phosphodiesterase inhibitor (PDE5) sildenafil, the synthetic analog of prostacyclin iloprost, and calcium channel blockers (CCB).

For the drug survival analysis, a subcohort of patients, with regular follow-up every 3–4 months in a single centre, for whom detailed medical information regarding exact date of each drug initiation and discontinuation and reason for discontinuation was available, was studied. Reasons for drug discontinuation were classified as associated toxicity, lack of response, disease stabilization, or miscellaneous. Kaplan–Meier curves were plotted to determine continuation rates for the different immunosuppressive/antiproliferative and vasoactive drugs separately and were compared with the log-rank test. Discontinuation of treatment (for any of the reasons described above) was considered an event. Comparisons among groups for continuous variables were made by *t* test and for categorical variables by the *χ*^2^ test. Statistical significance was assumed for values of *p* < 0.05. All statistical analyses were performed in SPSS version 24.0.

## Results

### Treatment patterns

A total of 497 patients (438 women, 243 diffuse SSc, mean disease duration 11.8 ± 8.4 years) were included in the study. Demographics and SSc clinical characteristics are presented in Table 1. Regarding immunosuppressive/antiproliferative treatments, a total of 362 patients was administered at least one of these agents at some point during the disease course. As shown in Table 2, MTX was the most frequent agent administered in 265 patients (51% of them with diffuse subtype) followed by CYC in 130 (75% diffuse), MMF in 58 (62% diffuse), AZA in 52 (52% diffuse), RTX in 43 (79% diffuse), and TCZ in 25 (56% diffuse) of patients, respectively. Overall, 222 out of 362 patients had received only 1 agent, 97 patients had received 2 different agents, 37 patients had received 3 agents, and 6 patients more than 3 agents.

**Table 1:** Comparable demographics and clinical characteristics of multicentre (*n* = 497) and single-centre (*n* = 181) systemic sclerosis cohorts

	Multicentre cohort *n* = 497 (243 diffuse)	Single-centre cohort* *n* = 181 (83 diffuse)
Age (years)	59.7 ± 13.9	55.8 ± 14.6
Diffuse	58.5 ± 14.2	54.3 ± 13.9
Limited	60.6 ± 13.8	56.8 ± 15.2
Gender (women)	438 (88%)	161 (89%)
Diffuse	213 (88%)	75 (91%)
Limited	225 (89%)	86 (86%)
Disease duration (years)	11.8 ± 8.4	11.8 ± 8.3
Diffuse	12.4 ± 8.0	11.9 ± 7.8
Limited	11.2 ± 8.4	11.9 ± 8.6
Arthritis	298 (60%)	114 (63%)
Diffuse	156 (64%)	58 (70%)
Limited	142 (56%)	56 (57%)
Pulmonary fibrosis	308 (62%)	119 (66%)
Diffuse	190 (78%)	66 (80%)
Limited	118 (46%)	53 (54%)
Pulmonary hypertension	54 (11%)	18 (10%)
Diffuse	30 (12%)	9 (11%)
Limited	24 10%)	9 (10%)
Cardiac rhythm disorders	68 (14%)	24 (13%)
Diffuse	40 (16%)	13 (15%)
Limited	28 (11%)	11 (11%)
Renal crisis	9 (2%)	5 (3%)
Diffuse	6 (2%)	3 (3%)
Limited	3 (1%)	2 (2%)
Digital ulcers	268 (54%)	96 (53%)
Diffuse	168 (69%)	53 (64%)
Limited	100 (39%)	43 (44%)
Upper gastrointestinal involvement	363 (73%)	136 (75%)
Diffuse	207 (85%)	67 (83%)
Limited	156 (61%)	66 (68%)

Data are shown as mean ± 1 standard deviation and as numbers (percentage) *p > 0.05 for all comparisons between multicentre and single centre cohort

**Table 2:** Immunosuppressive/antiproliferative and vasoactive agents administered anytime during disease course in a multicentre systemic sclerosis cohort

	Total (*n* = 497)	Diffuse (*n* = 243)	Limited (*n* = 254)	*p*
Immunosuppresives/ antiproliferatives				
Methotrexate	265 (53%)	134 (55%)	131 (52%)	NS
Cyclophosphamide	130 (26%)	97 (40%)	33 (13%)	0.021
Mycophenolate	58 (12%)	36 (15%)	22 (9%)	0.068
Azathiorine	52 (10.5%)	27 (11%)	25 (10%)	NS
Rituximab	43 (9%)	34 (14%)	9 (4%)	0.001
Tocilizumab	25 (5%)	14 (6%)	11 (4%)	NS
Vasoactives				
Calcium channel blockers	338 (68%)	172 (71%)	166 (65%)	NS
Bosentan	164 (33%)	104 (43%)	60 (24%)	0.001
Abrisentan	13 (3%)	7 (3%)	6 (2%)	NS
Macitentan	9 (2%)	3 (1%)	6 (2%)	NS
Sildenafil	34 (7%)	23 (9%)	11 (4%)	0.05
Iloprost	33 (7%)	18 (7%)	15 (6%)	NS
Only immunosuppressive treatment	209 (42%)	96 (40%)	113 (45%)	NS
Only vasoactive treatment	39 (8%)	20 (8%)	19 (7%)	NS
Both treatments	156 (31%)	102 (42%)	54 (21%)	0.001
No treatment	93 (19%)	25 (10%)	68 (27%)	0.001

All data are shown as numbers (percentage)

Mean disease duration: 11.8 ± 8.4 years, (mean + standard deviation)

Regarding vasoactive agents, 338 out of 497 patients had ever received CCB. For other than CCB, i.e., advanced vasoactive agents such as ERAs, iloprost, and sindenafil, 195/497 patients had received them during the disease course. Among these agents, ERAs were the most frequently dispensed in 186 patients (61% diffuse) followed by sildenafil in 34 (66% diffuse) and iloprost in 33 patients (55% diffuse) (Table 2). Among those 195 patients, 138 had received only ERAs, 4 only sildenafil, 7 patients only iloprost, 22 patients received combination of ERAs and sildenafil, 18 combined treatment with ERAs and iloprost, and 8 patients received combination of ERAs, sildenafil, and iloprost. Overall, among 48 patients who received combined therapy, 26 patients presented PAH and 22 refractory DUs.

Of note, 27% of all SSc patients had never been treated with immunosuppressive/antiproliferative agents, 61% had never received advanced vasoactive therapy, and 19% of all patients were never administered either treatment (Table 2). Moreover, 23% of patients developing PF had never received immunosuppressive/antiproliferative treatment while among patients with DUs, 33% of them had never received advanced vasoactive drugs.

### Drug survival analysis

The subcohort which was used for the drug survival analysis comprised 181 SSc patients and was representative of the entire multicentre cohort regarding demographics and clinical characteristics (Table 1).

**Fig. 1:**
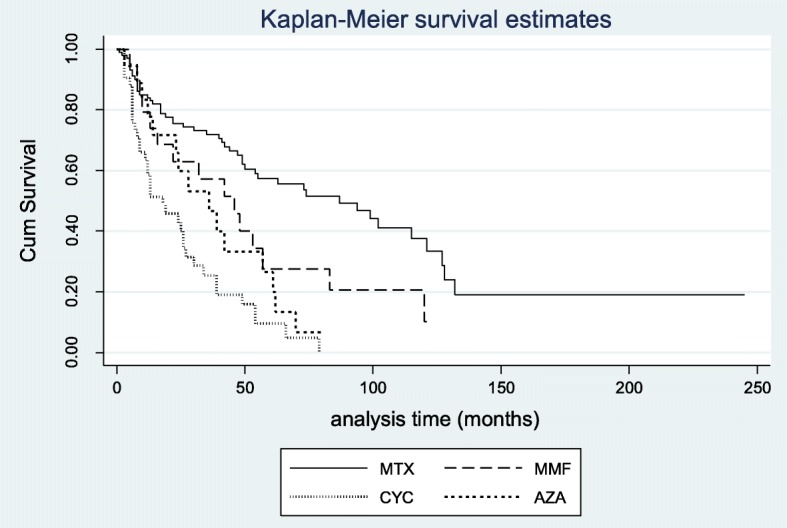
Kaplan–Meier curves showing drug survival of immunosuppressive/antiproliferative agents in 181 patients with systemic sclerosis. Mean disease duration: 11.8 ± 8.4 years, mean + SD. AZA = azathioprine, CYC = cyclophosphamide, MMF = mycophenolate mofetil, MTX = methotrexate

Regarding immunosuppressive/antiproliferative agents, survival of MTX was 91% after 6 months, dropped to 84% after 1 year from treatment initiation and reached 72% after 36 months. CYC exhibited significantly lower survival rates compared to MTX (59% at 12 months, 43% at 24 months, and 26% after 36 months from treatment initiation) (Table 3 and Fig. 1). Survival was also significantly lower for MMF and AZA compared to MTX at 12, 24, and 36 months after treatment initiation. Regarding RTX and TCZ, the small number of cases and the paucity of long-term data precluded their inclusion in the analysis. However, it should be noted that for TCZ, which was used in the context of common clinical practice and not under any long-term clinical trial, after 12 months 10 out of 13 patients who entered this treatment continued on this regimen.

**Table 3:** Survival rates (95% confidence intervals) of immunosuppressive/antiproliferative and vasoactive agents in 181 patients with Systemic sclerosis

Immunosuppressives	Methotrexate *	Cyclophosphamide **	Mycophenolate***	Azathioprine
6 months	0.91 (0.84–0.95)	0.76 (0.60–0.86)	0.95 (0.70–0.98)	0.88 (0.62–0.97)
12 months	0.84 (0.75–0.90)	0.59 (0.42–0.72)	0.74 (0.48–0.88)	0.78 (0.51–0.91)
24 months	0.75 (0.66–0.83)	0.43 (0.27–0.58)	0.63 (0.40–0.80)	0.60 (0.34–0.78)
36 months	0.72 (0.62–0.80)	0.26 (0.13–0.40)	0.53 (0.32–0.74)	0.47 (0.23–0.67)
48 months	0.65 (0.54–0.74)	0.16 (0.06–0.30)	0.40 (0.20–0.61)	0.33 (0.12–0.54)
Vasoactives	Endotelin-receptor antagonists^**+**^	Sildenafil^**++**^	Calcium channel blockers	–
6 months	0.92 (0.84–0.96)	0.93 (0.69–0.99)	0.99 (0.93–0.99)	–
12 months	0.88 (0.78–0.94)	0.8 (0.52–0.93)	0.97 (0.91–0.99)	–
24 months	0.86 (0.76–0.92)	0.8 (0.52–0.93)	0.91 (0.85–0.96)	–
36 months	0.84 (0.73–0.91)	0.70 (0.41–0.88)	0.87 (0.78–0.92)	–
48 months	0.79 (0.66–0.88)	0.70 (0.41–0.88)	0.83 (0.74–0.89)	–

*0.001 vs. CYC, 0.039 vs. MMF, 0.001 vs. AZA

**0.009 vs. MMF, 0.075 vs. AZA *** 0.337 vs. AZA

^**+**^ 0.256 vs. sildenafil, 0.562 vs. CCB ^**++**^ 0.187 vs. CCB

Methotrexate and tocilizumab were administered for skin sclerosis or arthritis. Cyclophosphamide, mycophenolate, azathioprine, and rituximab were administered mainly for pulmonary fibrosis. Endothelin receptor antagonists (ERAs) and sildenafil were administered either for digital ulcers or pulmonary arterial hypertension and calcium channel blockers (CCB) were administered as first-line vasodilators

**Fig. 2:**
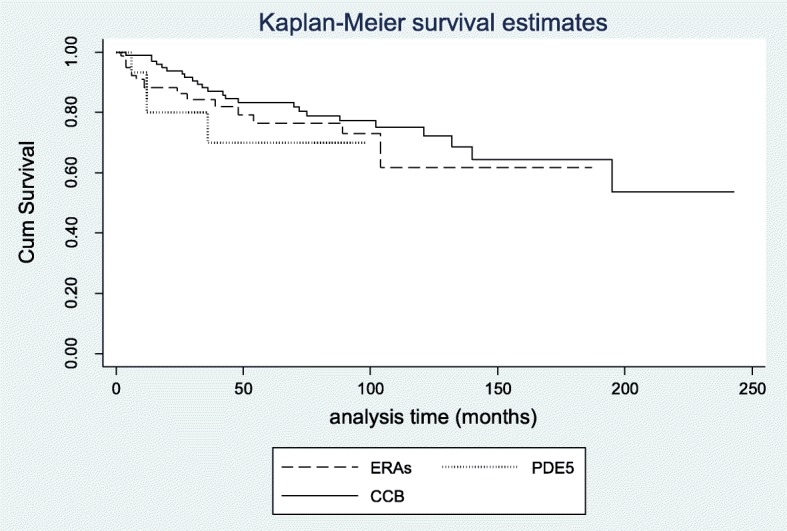
Kaplan–Meier curves showing drug survival of vasoactive agents in 181 patients with systemic sclerosis. Mean disease duration: 11.8 ± 8.4 years, mean + SD. CCB = calcium channel blockers, ERAs = endothelin receptor antagonists, PDE5 = 5-phosphodiesterase inhibitor

Among vasoactive drugs, CCB, ERAs, and PDE5 inhibitors presented high and comparable retention rates (Table 3 and Fig. 2). Specifically, survival of ERAs was 92% after 6 months, dropped to 88% after 1 year and reached 82% after 36 months. For sildenafil, the survival rate was 93% at 6 months, decreased to 80% at 12 and 24 months, and in the third year reached 71%. Analysis according to different indications for ERAs and sildenafil revealed comparable rates after 12, 24, and 36 months for ERAs (90%, 87%, and 82% in case of DUs vs. 93%, 88%, and 84% in case of PAH, *p* = 0.895) and sildenafil (84% for all timepoints in case of DUs vs. 89%, 78%, and 78% in case of PAH, respectively, *p* = 0.475). For CCB the survival rates were 97%, 91%, and 87% after 12, 24, and 36 months from treatment initiation. Finally, for iloprost, which was mostly given as salvage treatment, the respective rates at the end of 6, 12, and 24 months were 91%, 41%, and 31%.

Regarding drug discontinuation (Table 4), the most frequent cause for all immunosuppressive/antiproliferative drugs was inefficacy and accounted for 41% of all dropouts in the MTX group, 31% in CYC, and 33% in MMF. Adverse events were responsible for 30% of all MTX, 26% of CYC, and 27% of all MMF discontinuations. Interestingly, drug cessation after disease stabilization was noted in 9.5% of all MTX cases, 21% of all CYC, and 15% of all MMF cases.

**Table 4:** Reasons for discontinuation of immunosuppressive/antiproliferative and vasoactive agents in 181 patients with systemic sclerosis

	Inefficacy	Adverse event	Disease stabilization	Miscellaneous
Methotrexate (*n* = 49/106)*	20 (41%)	15 (30%)	10 (21%)	4 (8%)
Cyclophospamide (*n* = 35/48)	11 (31%)	9 (26%)	10 (29%)	5 (14%)
Mycophenolate (*n* = 18/27)	6 (33%)	5 (27%)	4 (22%)	3 (22%)
Azathioprine (*n* = 13/16)	7 (54%)	3 (23%)	–	3 (23%)
Rituximab (*n* = 3/6)	1 (33%)	1 (33%)	–	1 (33%)
Tocilizumab (*n* = 3/13)	1 (33%)	1 (33%)	–	1 (33%)
ERAs (*n* = 17/83)	2 (12%)	12 (71%)	–	3 (17%)
Bosentan (*n* = 14/71)	1 (7%)	10 (71%)	–	3 (21%)
Abrisentan (*n* = 2/7)	1 (50%)	1 (50%)	–	–
Macitentan (*n* = 1/5)	–	1 (100%)	–	–
Iloprost (*n* = 8/12)	1 (13%)	4 (50%)	–	3 (37%)
Sildenafil (*n* = 3/15)	1 (33%)	2 (66%)	–	–

*n of agents discontinued/*n* of agents administered

For the group of vasoactive agents, the most frequent cause of discontinuation was the presence of adverse events ranging from 50% for iloprost to 71% for ERAs while inefficacy was responsible for 12% of treatment cessation in the ERAs group, 13% in iloprost, and 33% in sildenafil (Table 4).

## Discussion

To the best of our knowledge this is the first study to analyse the survival of immunosupressive/antiproliferative and vasoactive regimens used in SSc based on real-world data.

The analysis in the multicentre cohort revealed that MTX was the most commonly administered immunosuppressive/antiproliferative agent in SSc patients followed by CYC, MMF, and AZA while a small number of patients was treated with biologics (RTX or TCZ) for which observational studies have reported promising results from their use in SSc [8–11]. This results can be explained, as MTX, based on randomized clinical trials (RCTs) and observational studies [12–14], is efficient in skin sclerosis and arthritis, the two most frequent manifestations of SSc and currently MTX is included in the EULAR recommendations for skin disease [15], representing the first choice of treatment of most physicians for mild to moderate cases of SSc. On the other hand, CYC and MMF which are considered more potent are recommended for the severe complications of SSc, like progressive PF or cardiac involvement [15–19].

Regarding vasoactive treatments, CCB, an old and conventional group of vasodilators, were the most frequently administered in SSc patients. Among more advanced vasoactive agents, ERAs were the treatment of choice in the majority of cases. In our study, the percentage of sildenafil- and iloprost-treated patients was significantly lower compared to other large published SSc cohorts [20, 21]. A possible explanation is that these two drugs are not approved by the national insurance system for the management of digital vasculopathy in Greece and their prescription requires special permission from a national committee, thus limiting their ordinary use. Of note, 25% of patients (48% among patients with PAH) who needed advanced vasodilation received double or triple combination therapy. The latter is in accordance with the results of other studies [22, 23] and during the last years, there is growing evidence that combined therapy compared to monotherapy results in improved functional outcomes both for PAH [24–26] and digital vasculopathy [27].

An interesting finding of the present study was that 23% of patients with PF and 33% with DUs never received immunosuppressive/antiproliferative or advanced vasoactive agents, respectively, during the entire disease course, possibly reflecting the group of patients presenting a mild disease pattern. In a recent retrospective study of 151 SSc patients suffering from PF [28], analysis based on a watchful waiting decision model showed that among patients with mild lung involvement, survival was higher in the untreated group. Additionally, in the ESOS study of 2017 [29], untreated patients had comparable disease outcomes and survival rates to treated patients with MTX, CYC, or MMF. Taking into account that in both studies untreated patients suffered from milder disease, these results and the results of our study indicate that aggressive treatment may not always be needed in case of mildly manifested complications, sparing in many cases patients from unnecessary toxic treatments. On the other hand, it is possible that in real-world some patients might be undertreated due to inadequate follow-up or underestimation of disease severity. A data analysis in 3248 patients of the German national registry revealed that 30% of patients with DUs and 20% with PAH never received vasoactive drugs [20] while recently a EUSTAR analysis of 1800 patients revealed the same for 25% of patients with DUs [21], with both studies questioning the adequate management of these patients.

Regarding drug survival, analysis in one centre cohort, representative of the entire multicentre cohort, revealed that MTX presented the longest mean overall drug survival followed by MMF, AZA, and CYC with lack of efficacy or adverse events being responsible for drug cessation in around 60 to 70% of cases. However, these results cannot be directly compared, firstly, because MTX is usually administered for milder manifestations and secondly because CYC and to a lesser degree MMF are typically administered as induction therapies and consequently after 12–18 months are replaced by less-toxic maintenance therapies. It should be noted that in our study a small proportion of patients received per os CYC for 36 or even 48 months, beyond the usual induction drug load, and these patients correspond to a former period when therapeutic choices were limited and prolonged per os CYC treatment was necessary. In the literature, the reported discontinuation rates vary from 30 to 45% for CYC [17, 19, 30] and from 10 to 40% for MMF after 12 to 24 months of treatment [18, 31, 32] while another concerning issue is the results from studies reporting recurrence of the disease after CYC or MMF cessation [33, 34]. Altogether, the mediocre retention rates of CYC and MMF and the risk of disease relapse after treatment cessation are indicative of the therapeutic predicament that physicians often confront in daily clinical practice and of the need for newer more effective long-term treatments. Interestingly, in our study despite the short follow-up for TCZ treatment, 80% of patients continued TCZ after 12 months of treatment which could be indicative of its potential efficacy in SSc. Certainly, RCTs are needed to clarify the position of TCZ and other biologics like RTX in the treatment of SSc.

For vasoactive agents, the results were quite different, revealing high and comparable retention rates for CCB, ERAs, and PDE5 inhibitors and limited numbers of cases who had to stop treatment mainly due to adverse events. These results are confirmed in other observational studies [35–37] and indicate that contrary to the unmet need of adequate immunomodulation in SSc, vasculopathy seems to be managed in a more efficient way after the introduction of advanced vasoactive regimens during the last 15 years.

Our study has certain limitations. Firstly, although analysis of treatment patterns was performed in the multicentre cohort, the lack of detailed data for drug initiation and/or cessation in the other cohorts made the drug survival analysis feasible only in one single-centre cohort. It should be noted however that this subcohort was demographically and clinically representative of the entire multicentre cohort possibly allowing to extrapolate the results to the multicentre cohort. Additionally, the retrospective nature of the study and the lack of clinical data for all patients in the multicentre cohort forbade the comparison between treated and untreated patients in order to address differences between the two groups. Finally, as in all multicentre cohorts, treatment decisions vary among physicians and a possible influence of this heterogeneity on the results cannot be ignored.

## Conclusions

To conclude, the results of the present study indicate that a considerable proportion of SSc patients may be either over- or under-treated, thus making risk stratification and individualized evidence-based treatment mandatory in daily clinical practice. Additionally, despite the advances in understanding the disease mechanisms, the retention rates of immunosuppressive/antiproliferative agents are currently low, suggesting a mediocre efficacy which explains the lack of improved survival of patients with SSc. Although vasculopathy seems to be managed more rigorously, it is clear that our efforts should focus on the discovery of more effective and targeted disease-modifying agents for these patients.

## Abbreviations

AZA: Azathioprine
CCB: Calcium channel blockers
CT: Computed tomography
CYC: Cyclophosphamide
DUs: Digital ulcers
ERAs: Endothelin receptor antagonists
MMF: Mycophenolate mofetil
MTX: Methotrexate
NSIP: Non-specific interstitial pneumonia
PAH: Pulmonary arterial hypertension
PAP: Pulmonary artery pressure
PDE5: 5-Phosphodiesterase inhibitor
PF: Pulmonary fibrosis
RTX: Rituximab
SSc: Systemic sclerosis
TCZ: Tocilizumab
UIP: Usual interstitial pneumonia
